# Dietary effects of organic selenium and zinc combination on *in vitro* microbial dynamics in the rumen

**DOI:** 10.5455/javar.2025.l942

**Published:** 2025-08-18

**Authors:** Moh Sofi’ul Anam, Budi Prasetyo Widyobroto, Andriyani Astuti, Gunawan Gunawan, Ali Agus

**Affiliations:** 1 Department of Animal Nutrition and Feed Science, Faculty of Animal Science, Universitas Gadjah Mada, Yogyakarta, Indonesia; 2 Department of Animal Production, Faculty of Animal Science, Universitas Gadjah Mada, Yogyakarta, Indonesia; 3 Research Center for Animal Husbandry, National Research and Innovation Agency (BRIN), Bogor, Indonesia

**Keywords:** Microbial composition, selenium, trace mineral, Zinc

## Abstract

**Objective:**

This study used an *in vitro* approach to investigate the effects of combined organic selenium and zinc supplementation on rumen microbial communities.

**Materials and Methods:**

A completely randomized design was employed with five dietary treatments: basal diet only (CON), CON + selenium 0.30 ppm (part per million) + zinc 60 ppm (SZ-1), CON + selenium 0.45 ppm + zinc 60 ppm (SZ-2), CON + selenium 0.30 ppm + zinc 90 ppm (SZ-3), and CON + selenium 0.45 ppm + zinc 90 ppm (SZ-4). Selenium and zinc were provided in the form of organic-chelated methionine.

**Results:**

Analysis of rumen microbiota through *16S rRNA* gene sequencing showed no significant differences in microbial diversity (*p* > 0.05); however, microbial composition was significantly affected. SZ-2, SZ-3, and SZ-4 groups exhibited an increased prevalence of *Bacteroidetes* and *Proteobacteria* and a reduction in *Firmicutes* compared to CON and SZ-1 (*p* < 0.05). The relative abundance of *Patescibacteria* and *Euryarchaeota* was also reduced in the SZ-2, SZ-3, and SZ-4 groups (*p* < 0.05). At the genus level, *Prevotella*, *Ruminococcus*, and *Rikenellaceae RC9 gut* groups were enriched in SZ-2, SZ-3, and SZ-4, whereas *Lachnospiraceae XPB1014* and *Christensenellaceae R-7* group decreased (*p* < 0.05).

**Conclusion:**

Combined selenium and zinc supplementation as organic trace minerals significantly modulates rumen microbial composition, enhancing the relative abundance of carbohydrate-degrading bacteria while reducing methanogen-related taxa.

## Introduction

Trace minerals (TMs), commonly referred to as microelements, such as selenium and zinc, are recognized for their crucial role in metabolic function, productivity, and the overall health of livestock [1–3]. In addition to their metabolic functions, TM also influences the rumen environment, as microorganisms depend on these minerals for protein synthesis and fermentation activity [4, 5]. For instance, selenium is an essential component of selenoproteins and antioxidant enzymes such as glutathione peroxidase, which safeguard cells against the harmful impacts of free radicals generated during lipid oxidation [6]. Similarly, zinc is crucial for the catalytic activity of over 200 enzymes, many of which possess robust antioxidant capabilities, including superoxide dismutase, helping to protect cells from damage caused by reactive oxygen species [7].

Ruminant animal feed primarily originates from forage, which often has varying TM content depending on climatic conditions, agricultural practices, soil composition, and plant type [8]. Since the body cannot synthesize TM independently, the TM content in the feed, including selenium and zinc, can be supplemented through the diet to maintain optimal production performance [9]. Unlike monogastric animals, ruminants rely on the rumen‘s microbial population and digestive enzymes for nutrient breakdown [10].

The rumen microorganisms constitute diverse types, including fungi, bacteria, protozoa, viruses, and archaea. These microorganisms are necessary for fermentation, which converts fibrous materials and plant cell walls into molecules that can be absorbed, especially proteins and volatile fatty acids [11]. Through anaerobic fermentation, these microbes play a crucial role in enhancing the productivity of ruminants and meeting most of the host’s energy needs. Several studies have indicated that TM can improve antioxidant status, increase microbial growth and fermentation, and enhance livestock development and yield [12–14]. For example, selenium supplementation has been shown to influence the relative abundance of multiple ruminal microbiotas, promoting certain fibrolytic bacteria in Barki sheep [15]. In Tibetan sheep, a diet supplemented with selenium significantly impacted the abundance of different bacterial groups [16]. Additionally, the comparative abundance of certain rumen bacteria was altered in groups supplemented with zinc [5].

Organic TM has gained attention in the last decade due to its greater safety, lower toxicity, and higher bioavailability than inorganic versions [16]. While several studies have explored the effects of selenium or zinc supplementation individually on rumen microbiota, research on their combined effects, particularly in organic forms, remains limited. Selenium and zinc are vital for microbial enzymatic processes and possess synergistic potential that could amplify their benefits in the rumen environment. A recent study by Anam et al. [17] demonstrated that combining organic selenium and zinc significantly enhances ruminal enzyme activity under *in vitro* conditions. These findings provide a foundation for further exploration, particularly in practical settings, to validate and expand upon the observed impacts on microbial dynamics. This study seeks to address these gaps by investigating the effects of combined organic selenium and zinc supplementation on microbial dynamics in the rumen.

While several studies have explored the effects of selenium or zinc supplementation individually on rumen microbiota, limited research has investigated the combined effects of these elements, especially in their organic forms. This combination is particularly significant, as selenium and zinc play crucial roles in supporting rumen microbial populations, and their combined effects on microbial community dynamics remain an important area for further exploration. Given the limited understanding of the interaction between these organic forms of TM in the rumen, this study aims to bridge this gap by investigating the effects of combined organic selenium and zinc supplementation on microbial dynamics in the rumen.

## Materials and Methods

### Ethical approval

Universitas Gadjah Mada‘s Institutional Animal Care and Use Committee in Indonesia gave permission for this work (025/EC-FKH/Eks./2023).

### Preparation of experimental diets

Table 1 displays the chemical makeup and composition of the basal diets. The chemical contents of feed samples were analyzed using the methodology outlined by AOAC [18]. The dry matter content was selected by subjecting the sample to oven drying at a temperature of 105°C until a constant mass was attained (#934.01). A Soxhlet extractor (#920.39) was used to evaluate the lipid content, while a Kjeldahl nitrogen analysis (#976.05) was used to measure the crude protein content. The sample was burned in a muffle furnace to determine the amount of ash present (#942.05). The amounts of acid and neutral detergent fiber were measured using a fiber analyzer (ANKOM Technology, New York, USA). Using inductively coupled plasma mass spectrometry (ICP-MS, Agilent 7850, Agilent, Santa Clara, USA), the quantities of selenium and zinc content in the feed were measured.

**Table 1.** Composition and nutrient levels of basal substrate (dry matter basis).

Items	Value (%)
Ingredients	
Elephant grass	59.70
Wheat bran	9.95
Corn	4.98
Rice bran	4.98
Palm kernel meal	12.94
Soybean meal	6.97
Mineral premix	0.50
Total	100.00
Nutrient levels	
Dry matter	89.78
Crude protein	14.78
Crude fat	3.44
Ash	8.21
Acid detergent fiber	34.56
Neutral detergent fiber	52.11
Selenium (ppm)	0.01
Zinc (ppm)	18.12

Description: Mineral premix (contains per kg): vitamin A 200,000 IU; vitamin D, 80,000 IU; vitamin E, 200 IU; Ca, 243.4 gm; P, 3.2 gm; K, 277.9 gm; Mg, 1.8 gm; Na, 24.3 gm; S, 130.4 mg; Fe, 12.5 mg; Mn, 1.2 mg; Cu, 179.4 mg; Co, 5.4 mg; I, 1.2 mg.

### Experimental treatments

The investigation used a complete randomized block design, focusing mainly on the treatment‘s impact. A total of five experimental treatments were adopted in the following manner: i) basal diet only (CON), ii) CON + selenium 0.30 ppm + zinc 60 ppm (SZ-1), iii) CON + selenium 0.45 ppm + zinc 60 ppm (SZ-2), iv) CON + selenium 0.30 ppm + zinc 90 ppm (SZ-3), and v) CON + selenium 0.45 ppm + zinc 90 ppm (SZ-4). Selenium and zinc were derived from commercial organic-chelated methionine (AminoxÒ, Fenanza Putra Perkasa Corp., Indonesia), with concentrations of 4,000 ppm and 15%, respectively. Organic selenium and zinc supplements were top-dressed over the basal substrate.

### In vitro rumen fermentation

The ruminal fluid was obtained from two Balinese cows provided with permanent cannulas (body weight, 320 ± 5 kg), which were fed an* ad libitum* diet consisting of forage and concentrate at a 60:40 ratio. The cows were fed twice daily for a minimum duration of 14 consecutive days with unrestricted access to clean water before collecting rumen fluid samples. Before the morning feeding, the rumen was properly obtained. Any leftover feed was then filtered out using two thicknesses of cheesecloth. Moreover, the laboratory received the rumen fluid, and it was added to a flask that had been heated to 39°C beforehand. A solution of McDougall’s buffer was prepared and introduced by carbon dioxide to create an oxygen-free environment while maintaining constant stirring using a magnetic stirrer at a temperature of 39°C [17]. After mixing the rumen fluid and buffer solution (1:4 v/v), 50 ml of inoculum was added to a 120 ml serum container with 500 mg of dry basal feed. The bottles were incubated at 39°C after being tightly sealed with a metal crimp and butyl rubber stopper. The outside of the bottles was then coated with shrink plastic. This study used six replications for each treatment and six blank samples with only 50 ml of rumen inoculum and no basal feed. After 48 h of incubation, the bottle was removed from the incubator and immersed in cold water to end the fermentation process. The rumen fluid and substrate residue were separated using a crucible fitted with glass wool.

### Ruminal bacteria DNA isolation and 16S ribosome-ribonucleic acid (rRNA) sequencing

Three randomly selected rumen liquid samples from each group were used for the molecular study using *16S rRNA* gene sequencing. Microbial DNA samples were extracted using the FavorPrepTM Soil DNA Isolation Mini Kit (Favorgen Biotech, Ping Tung, Taiwan) in accordance with the manufacturer’s instructions. A Qubit fluorometer (ThermoFisher) was utilized to assess the amount and quality of genomic DNA. The V3-V4 region of *16S rRNA* was replicated using 30 ng of genomic DNA from each sample and the universal primer sets 338F (5′-ACT CCT ACG GGA GGC AGC AG-3′) and 806R (5′-GGA CTA CHV GGG TWT CTA AT-3′). Furthermore, a unique eight-base barcode sequence was incorporated into each primer for sample identification purposes. Ideally, the S100 Thermal Cycler (Bio-Rad) and 2 × Phanta Max Master Mix (P515-01, Vazyme) were utilized for PCR-based amplification. Pre-denaturation at 95°C for 3 min was the first step in the PCR process. In addition, there were twenty-five 30-sec cycles of denaturation to unfold the molecular structure at 95°C, annealing for binding of complementary strands at 55°C, and extension of the DNA strand at 72°C. The last extension phase lasted 5 min at 72°C. All PCR products were cleared using Agencourt AMPure XP beads (A63880, Beckman Coulter), rinsed in the appropriate buffer, and then tagged to complete the construction of DNA nanoball (DNB) libraries. The concentration and size of the library were examined using the Agilent 2100 Bioanalyzer (Agilent, Palo Alto, California, USA). Afterward, on the DNBSEQ-G400 high-throughput sequencing platform, qualified DNB libraries were sequenced.

### Bioinformatics analysis

For further analysis, the FASTQ files were loaded into QIIME2 v9.2023, and primer sequence elimination was carried out using QIIME’s Cutadapt v2.6 plugin. Denoising of sequences was conducted with QIIME2’s DADA2 plugin. Moreover, the noise-reduced sequences were grouped into operational taxonomic units (OTUs) using the VSEARCH tool in QIIME’s feature-classifier plugin, which used the Silva database and a 97% similarity cut-off.

A Venn diagram was created utilizing the web-based Venn diagram creation tool (http://bioinformatics.psb.ugent.be/webtools/Venn/) to identify the OTUs common among different groups. The OTU table generated by QIIME2 was submitted to MicrobiomeAnalyst (https://www.microbiomeanalyst.ca/) and subsequently adapted, along with metadata, to the MicrobiomeAnalyst format for further analysis. Alpha-biodiversity indices, including abundance-based coverage estimator (ACE), abundance-based estimator of species richness (Chao1), Shannon, and Simpson, were statistically evaluated using analysis of variance (ANOVA). Beta diversity was evaluated through principal coordinates analysis (PCoA) with the Bray-Curtis distance matrix. The variations in beta-diversity distances were evaluated using permutational multivariate ANOVA (PERMANOVA). Furthermore, distinct microbial taxa were found at the genus taxonomy level by employing the linear discriminant analysis (LDA) effect size technique (LEfSe) with a significant threshold set at LDA > 4.0 and *p* < 0.05.

### Statistical analysis

Using the Statistical Package for the Social Sciences (SPSS 26.0, Chicago, IL), a one-way ANOVA was run on the data. Duncan’s multiple comparison test was used to determine whether there were significant differences between the treatments; values of *p* < 0.05 were considered statistically significant. The pooled SEM was displayed with the means of the data.

## Results

### Diversity of ruminal microbiota composition

**Figure 1.**
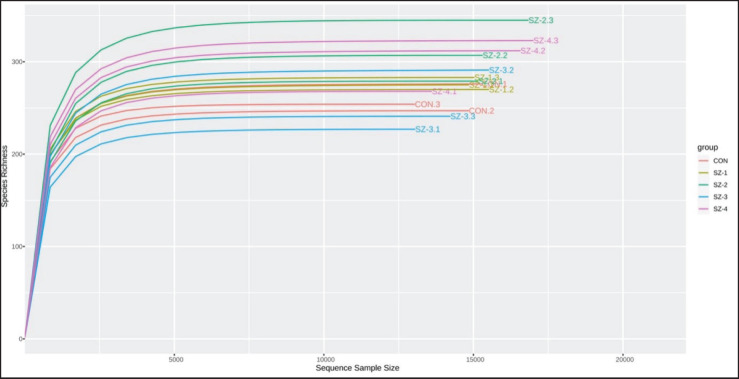
Rarefaction curves across the dietary treatment.

A total of 1,251,738 reads were acquired from the *16S rRNA* gene sequencing; 1,169,319 high-quality sequences were recovered for analysis following filtering, denoising, merging, and eliminating chimeras. On average, each sample had 77,955 ± 478 clean reads, and the Good‘s coverage, as determined by the detected OTUs, was found to be 99% (data not shown). Furthermore, as shown in Figure 1, the rarefaction curves nearly reached saturation, indicating that the sequencing depth was adequate to capture most of the microbiological data in the sample.

**Figure 2.**
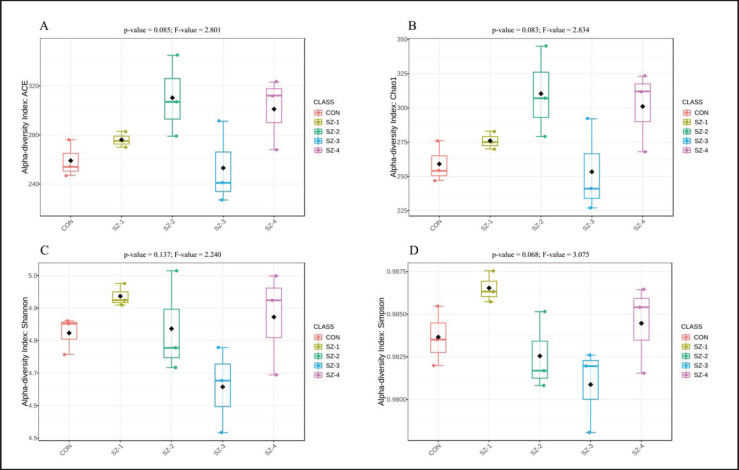
Alpha-diversity measurements of the rumen microbiota. A = ACE index, B = Chao1 estimates, C = Shannon diversity, D = Simpson diversity.

**Figure 3.**
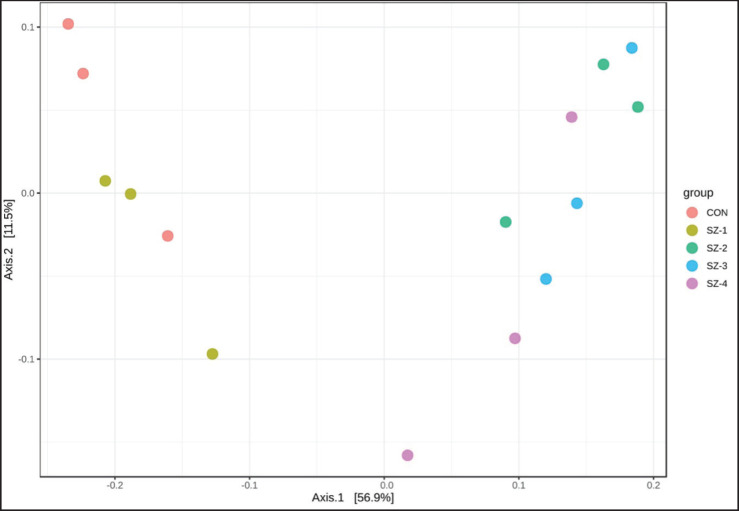
Measures of beta diversity across the diet groups at the OTUs level were plotted in the PCoA in 2D. Differences in the beta-diversity were tested by the PERMANOVA; Bray–Curtis distances were used to explain beta diversity across diet groups. PERMANOVA *F*-value = 4.990; *R^2^* = 0.666; *p*-value = 0.002.

This study set out to ascertain the effects of organic selenium and zinc feeding on the alpha and beta diversity of the rumen microbiota in five different groups. The alpha-diversity investigation found no statistically significant differences among the ACE, Chao1, Shannon, and Simpson indices (Fig. 2). The beta diversity of the microbial communities within the rumen groups was evaluated by combining PCoA with Bray–Curtis dissimilarity measurements. The results showed that the SZ-2, SZ-3, and SZ-4 groups had a distinct separation from the CON and SZ-1 groups (Fig. 3).

### Abundance and composition of ruminal microbiota taxa

**Figure 4.**
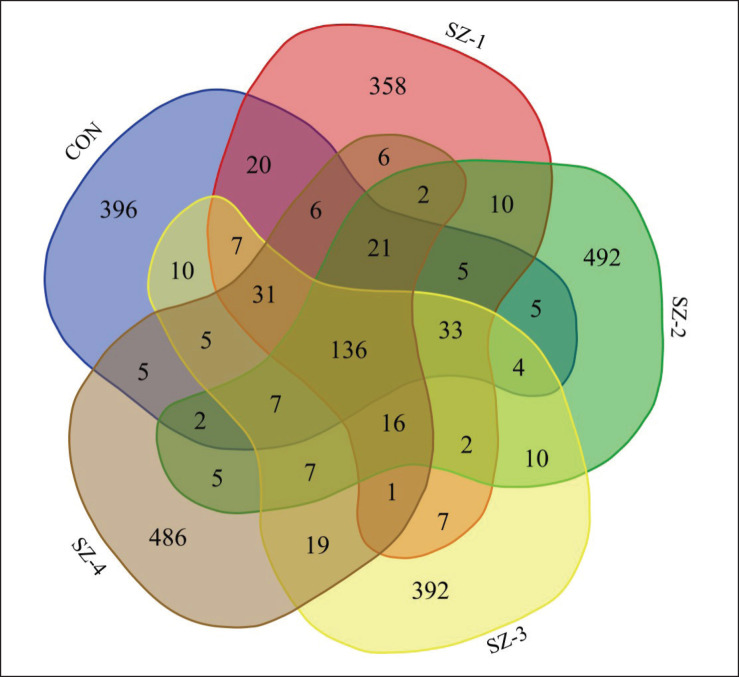
Venn diagram illustrating the overlap of the number of OTUs identified in the microbiota among dietary treatments.

**Figure 5.**
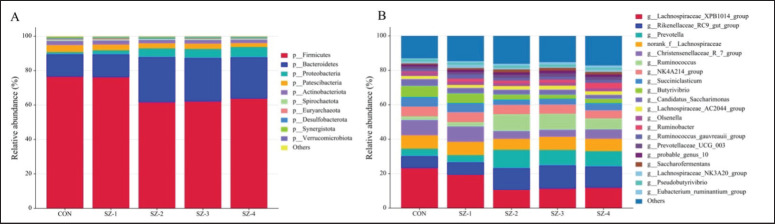
Histograms displaying the composition of rumen microbiota at the phylum (A) and genus levels (B). The horizontal axis represented five experimental groups, while the vertical axis depicted the relative abundance of each taxonomic category.

Based on the 97% sequence identity found in all samples, 2,506 OTUs were identified; of these, 136 were shared by all five groups. Additionally, the numbers specific to each group were as follows: 396, 358, 492, 392, and 486 for CON, SZ-1, SZ-2, SZ-3, and SZ-4, respectively (Fig. 4). The bacteria population was predominantly composed of phylum *Firmicutes* (68.05%) and *Bacteroidetes* (20.28%), accounting for over 88% of the total phyla. *Proteobacteria*, *Patescibacteria*, and *Actinobacteriota* constituted more than 1% of the community, with proportions of 4.05%, 3.11%, and 2.21%, respectively. In addition, the bacteria phyla that accounted for less than 1% were *Spirochaetota* (0.55%), *Euryarchaeota* (0.39%), *Desulfobacterota* (0.37%), *Synergistota* (0.35%), and *Verrucomicrobiota* (0.32%) (Table 2, Fig. 5A). In the SZ-2, SZ-3, and SZ-4 groups, the relative abundance of *Bacteroidetes* and *Proteobacteria* dramatically improved (*p *< 0.05); however, the phylum *Firmicutes* greatly declined (*p* < 0.05) and reached the CON and SZ-1 groups. The presence of *Patescibacteria* and *Euryarchaeota* also decreased significantly in the SZ-2, SZ-3, and SZ-4 groups compared to CON (*p* < 0.05). However, the abundances of *Actinobacteriota*, *Spirochaetota*, *Desulfobacterota*, *Synergistota*, *Verrucomicrobiota*, and other bacterial phyla did not change significantly as a result of the dietary treatments (*p >* 0.05).

The top 20 dominating genera are displayed in Figure 5B and Table 3, with a relative abundance of more than 1% designating them as predominant. The dominant microbiota among these genera includes *Lachnospiraceae XPB1014 *group (15.30%), *Rikenellaceae RC9 gut *group (10.39%), *Prevotella *(7.53%), *norank_f__Lachnospiraceae* (7.29%), *Christensenellaceae R-7 *group (6.44%), *Ruminococcus* (5.86%), *NK4A214 *group (5.60%), *Succiniclasticum* (4.26%), *Butyrivibrio* (3.80%), *Candidatus_Saccharimonas* (3.11%), *Lachnospiraceae AC2044 *group (2.01%), *Olsenella *(1.83%), *Ruminobacter* (1.76%), *Ruminococcus gauvreauii group* (1.75%), *Prevotellaceae UCG-003* (1.72%), *probable genus 10* (1.32%), *Saccharofermentans* (1.29%), *Lachnospiraceae NK3A20 *group (1.26%), *Pseudobutyrivibrio* (1.15%), and *Eubacterium ruminantium *group (1.06%). The proportions of *Rikenellaceae RC9 gut *group, *Prevotella, Ruminococcus*, and *Prevotellaceae UCG-003* were greater in the SZ-2, SZ-3, and SZ-4 groups compared to CON and SZ-1, while the *Lachnospiraceae XPB1014 *group, *Christensenellaceae R-7 *group, *Butyrivibrio*, and *Ruminococcus gauvreauii *group were reduced (*p* < 0.05). The SZ-4 group had a particularly higher comparative abundance of *Ruminobacter* compared to CON (*p* < 0.05). Conversely, *Saccharofermentans* was most abundant in the SZ-2 group (*p* < 0.05). *Candidatus_Saccharimonas* had significantly reduced levels in the SZ-2, SZ-3, and SZ-4 groups (*p* < 0.05) compared to CON.

**Figure 6.**
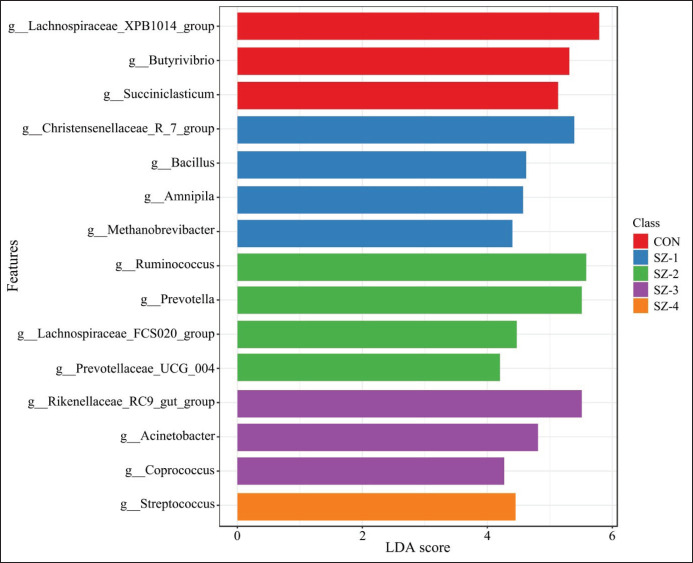
The LDA of effect size (LEfSe) between the diet groups at the genus level. The significant threshold was set at the LDA > 4.0 and *p* < 0.05.

With a threshold set at an LDA score > 4 and *p* < 0.05, LefSe analysis was used to find bacterial genera displaying significant differences between the five groups at the genus level (Fig. 6). According to the results, fifteen genera showed significant variations across the five groups. Specifically, the CON, SZ-1, SZ-2, SZ-3, and SZ-4 groups had three, four, four, three, and one different taxa, respectively. The CON group augmented the prevalence of *the Lachnospiraceae XPB1014* group, *Butyrivibrio*, and *Succiniclasticum*. The SZ-1 group had an augmentation in the *Christensenellaceae R-7* group, *Bacillus*, *Amnipila*, and *Methanobrevibacter*. We enhanced SZ-2 by adding *Ruminococcus*, *Prevotella*, *Lachnospiraceae FCS020* group, and *Prevotellaceae-UCG 004* abundance. SZ-3 intervention led to a rise in the *Rikenellaceae RC9 gut*, *Acinetobacter*, and *Coprococcus *groups, while SZ-4 specifically enhanced *Streptococcus*.

**Table 2.** Relative abundance (%) of bacteria phyla at each dietary treatment.

Items	Treatments					SEM	*p*-value
	CON	SZ-1	SZ-2	SZ-3	SZ-4		
*p__Firmicutes*	76.25^a^	76.17^a^	61.47^b^	62.08^b^	63.36^b^	2.03	0.001
*p__Bacteroidetes*	13.10^b^	13.22^b^	26.25^a^	25.20^a^	24.18^a^	1.67	< 0.001
*p__Proteobacteria*	1.36^b^	2.43^b^	5.21^a^	5.33^a^	6.11^a^	0.59	0.010
*p__Patescibacteria*	4.09^a^	3.29^ab^	2.92^bc^	3.04^bc^	2.30^c^	0.19	0.017
*p__Actinobacteriota*	2.42	2.40	2.04	2.18	2.04	0.07	0.185
*p__Spirochaetota*	0.57	0.55	0.54	0.58	0.53	0.02	0.972
*p__Euryarchaeota*	0.57^a^	0.63^a^	0.13^c^	0.34^b^	0.29^bc^	0.05	< 0.001
*p__Desulfobacterota*	0.41	0.32	0.44	0.36	0.34	0.02	0.295
*p__Synergistota*	0.44	0.34	0.36	0.31	0.31	0.03	0.548
*p__Verrucomicrobiota*	0.35	0.32	0.32	0.32	0.27	0.01	0.370
Others	0.44	0.33	0.32	0.27	0.27	0.02	0.089

Description: CON, basal diet only; SZ-1, CON + selenium 0.30 ppm + zinc 60 ppm; SZ-2, CON + selenium 0.45 ppm + zinc 60 ppm; SZ-3, CON + selenium 0.30 ppm + zinc 90 ppm; SZ-4, CON + selenium 0.45 ppm + zinc 90 ppm. SEM, standard error of the mean value. Mean values in the same row with different superscripts represent significant differences (*p* < 0.05).

## Discussion

This study investigated the synergistic effects of combined organic selenium and zinc supplementation on ruminal microbial composition, focusing on its potential to optimize fermentation efficiency and nutrient utilization. The findings revealed significant shifts in microbial profiles, emphasizing the novelty of combined organic selenium–zinc supplementation in modulating rumen fermentation pathways. The study found that the widely observed phyla were *Firmicutes* and *Bacteroidota*, which is in line with earlier findings [19]. These two phyla are made up of microbes that are essential to the ecology of ruminant animals and the operation of the rumen. The presence of *Firmicutes *and *Bacteroidetes*, which are obligate anaerobic bacteria, shows healthy rumen microbiota [20].

Numerous genes found in *Firmicutes* enable the synthesis of different digestive enzymes, which help animals digest and absorb nutrition. Conversely, *Bacteroidetes’* primary function is to break down proteins and carbohydrates and convert and acquire energy [21]. As a result, the rumen is anticipated to break down complex polysaccharides more quickly after TM is added [16, 22]. However, the observed increase in *Bacteroidetes* and concurrent decrease in *Firmicutes* in SZ-2, SZ-3, and SZ-4 groups highlight a novel microbial shift favoring carbohydrate metabolism and propionate production. The rise in the *Bacteroides* phylum was primarily due to an increase in various genera of the *Rikenellaceae RC9 gut* group, *Prevotella*, and *Prevotellaceae UCG-003*. On the other hand, the decrease in the *Firmicutes *phylum was mainly caused by a reduction in the *Lachnospiraceae XPB1014*, *Christensenellaceae R-7*, *Butyrivibrio*, and *Ruminococcus gauvreauii* group genera. The *Rikenellaceae RC9 gut* group significantly impacts lipid metabolism and the breakdown of rumen hemicellulose, while the *Prevotella* genus plays a role in breaking down as well as using complex carbohydrates and nitrogen compounds [23]. This compositional change, driven by the enrichment of *Prevotella* and *Rikenellaceae RC9 gut *group, underscores the potential of organic selenium–zinc supplementation to enhance fermentation efficiency [15]. However, the fibrolytic bacteria known as *Ruminococcus *belonging to the *Firmicutes* phylum, increased in population when organic selenium and zinc were administered.

**Table 3.** Relative abundance (%) of bacteria genera at each dietary treatment.

Items	Treatments					SEM	*p*-value
	CON	SZ-1	SZ-2	SZ-3	SZ-4		
*g__Lachnospiraceae_XPB1014_group*	23.07^a^	19.33^a^	10.64b	11.36b	11.87b	1.42	<0.001
*g__Rikenellaceae_RC9_gut_group*	6.84^b^	7.18^b^	12.55^a^	13.38^a^	12.29^a^	0.80	<0.001
*g__Prevotella*	4.50^b^	4.30^b^	10.81^a^	9.09^a^	9.14^a^	0.75	<0.001
*norank_f__Lachnospiraceae*	7.76	7.68	6.29	7.61	7.05	0.26	0.350
*g__Christensenellaceae_R_7_group*	8.92^a^	8.99^a^	4.58^b^	4.11^b^	5.49^b^	0.59	<0.001
*g__Ruminococcus*	1.96^c^	2.35^c^	9.62^a^	9.23^a^	6.25^b^	0.89	<0.001
*g__NK4A214_group*	5.86	5.97	6.68	5.58	4.98	0.17	0.430
*g__Succiniclasticum*	5.68	5.30	2.97	3.35	4.09	0.43	0.175
*g__Butyrivibrio*	6.18^a^	5.36^a^	2.72^b^	2.10^b^	2.43^b^	0.52	0.006
*g__Candidatus_Saccharimonas*	4.09^a^	3.29^ab^	2.92^bc^	3.04^bc^	2.30^c^	0.19	0.017
*g__Lachnospiraceae_AC2044_group*	1.82	1.73	2.14	2.32	2.08	0.09	0.215
*g__Olsenella*	1.93	1.95	1.73	1.82	1.74	0.05	0.422
*g__Ruminobacter*	0.79^b^	1.71^ab^	1.43^ab^	1.63^ab^	3.11^a^	0.31	0.200
*g__Ruminococcus_gauvreauii_group*	1.43^b^	2.39^a^	1.55^b^	1.70^b^	1.60^b^	0.11	0.023
*g__Prevotellaceae_UCG_003*	1.49^bc^	1.45^c^	1.87^a^	2.00^a^	1.83^ab^	0.07	0.019
*g__probable_genus_10*	0.99	1.30	1.36	1.58	1.34	0.08	0.215
*g__Saccharofermentans*	0.67^c^	1.011^bc^	1.69^a^	1.56^ab^	1.49^ab^	0.13	0.022
*g__Lachnospiraceae_NK3A20_group*	1.14	2.00	0.98	0.75	1.30	0.16	0.110
*g__Pseudobutyrivibrio*	1.29	1.12	1.10	1.15	1.10	0.03	0.288
*g__Eubacterium_ruminantium_group*	0.49	0.85	1.17	1.38	1.30	0.13	0.171
Others	13.08	14.72	16.22	15.25	17.21	1.92	0.059

Description: CON, basal diet only; SZ-1, CON + selenium 0.30 ppm + zinc 60 ppm; SZ-2, CON + selenium 0.45 ppm + zinc 60 ppm; SZ-3, CON + selenium 0.30 ppm + zinc 90 ppm; SZ-4, CON + selenium 0.45 ppm + zinc 90 ppm. SEM, standard error of the mean value. Mean values in the same row with different superscripts represent significant differences (*p* < 0.05).

The supplementation of organic selenium and zinc resulted in a considerable rise in the *Proteobacteria* proportion while decreasing *Patescibacteria* and *Euryarchaeota*‘s presence. Phylum *Proteobacteria* is a substantial assemblage of bacteria that engage in the process of fermenting carbohydrates into ethanol. This activity is significant in rumen metabolism, contributing to crucial functions, such as biofilm formation and fermentation [20]. The primary genus in the phylum was *Ruminobacter*, which had considerable growth when supplemented with selenium and zinc. *Ruminobacter* genus potentially contributes to variations in fermentation end products [20]. This study suggests that organic selenium–zinc may enhance nutrient capture and microbial attachment to feed particles, facilitating efficient nutrient utilization. Additionally, the reduction in methanogenic *Euryarchaeota* observed in selenium–zinc-supplemented groups suggests the potential environmental benefits of organic selenium–zinc supplementation. Methanogens play a central role in hydrogen utilization, and their reduced abundance indicates a shift in hydrogen flux toward propionate production. These findings align with previous research on selenium supplementation and its ability to mitigate methane emissions [24].

The LEfSe analysis offered additional information regarding microbial shifts induced by organic selenium–zinc supplementation. Genera such as *Ruminococcus*, *Prevotella*, and *Prevotellaceae UCG-004* were highly enriched in SZ-2-supplemented groups, emphasizing their functional roles in carbohydrate and fiber digestion. Moreover, *Ruminococcus* synthesizes cellulases and hemicellulases, enabling the breakdown of cellulose and hemicellulose into fermentable substrates [25]*.* Meanwhile, *Prevotella* contributes to the hydrolysis of carbohydrates and proteins, enhancing nitrogen utilization and feed efficiency [26]. *Prevotella* can decompose different types of complex carbohydrates, producing propionate, which serves as the main substrate for gluconeogenesis in the ruminants’ liver [27]. *Prevotellaceae UCG-004* functions in breaking down polysaccharides and producing branched-chain volatile fatty acids [28]. These functional roles underscore the practical implications of organic selenium–zinc supplementation for improving ruminant productivity, particularly in diets rich in fibrous materials.

The predominant genus *XPB1014* group from the *Lachnospiraceae* family is known for its capacity to convert starch in the diet [29]. This study found that the group belonging to the *Firmicutes *phylum decreased when selenium and zinc were added. In contrast, the *Rikenellaceae RC9 gut* group and *Prevotella* from the *Bacteroidetes *phylum increased following the supplementation treatment through extra feeding. Various studies have shown a higher presence of *Bacteroidetes* and greater production of volatile fatty acids [15]. These studies attribute the higher abundance of glycan-degrading enzymes during fermentation to *Bacteroidetes* rather than *Firmicutes *[30]. Compared to previous studies, this research represents a significant step forward in understanding the combined effects of selenium and zinc on ruminal microbiota. Tian et al. [24] demonstrated that selenium supplementation alone reduced methanogen populations, while Hendawy et al. [4] highlighted selenium’s role in enhancing volatile fatty acid production. This study expands on these findings by demonstrating the synergistic effects of selenium and zinc in enhancing carbohydrate-degrading genera, such as *Prevotella* and *Ruminococcus*, and reducing methanogenic populations. Additionally, the observed enrichment of *Ruminobacter* and the reduction of methanogens suggest a dual benefit of organic selenium–zinc supplementation: optimizing fermentation efficiency and mitigating methane emissions. These results align with Rabee et al. [15], who observed that selenium supplementation, particularly in the form of bio-nanostructured selenium, significantly improved rumen fermentation dynamics and altered the microbial community composition in lactating sheep, emphasizing its role in enhancing nutrient digestibility and reducing methanogen populations.

Although this study offers important insights into the modulation of ruminal microbiota through organic selenium–zinc supplementation, it is important to recognize certain limitations. Primarily, the *in vitro* design may not entirely reflect the complexities of an *in vivo* rumen environment, where factors such as host interactions and feed variability have significant influence. Second, the long-term impacts of organic selenium–zinc supplementation on microbial stability, animal performance, and health outcomes remain unexplored. Future studies should focus on *in vivo* experimentation to validate these findings under practical feeding conditions, particularly in animals with TM deficiencies. Additionally, investigating the interplay between microbial shifts and fermentation end products will provide a more comprehensive understanding of selenium and zinc supplementation‘s effects on rumen metabolism.

## Conclusion

In conclusion, dietary organic selenium and zinc supplementation influenced the ruminal microbial abundance. *Bacteroidetes* and *Proteobacteria* showed a significant increase in relative abundances at the phylum level, while *Firmicutes*, *Patescibacteria*, and *Euryarchaeota* exhibited a notable reduction. At the genus level, there were increases in *Prevotella*, *Ruminococcus*, and *Prevotellaceae UCG-003*, along with decreases in the *Lachnospiraceae XPB1014* group, *Christensenellaceae R-7* group, *Butyrivibrio*, and *Ruminococcus gauvreauii *group. Based on the findings, a lower dose of organic selenium and zinc supplementation (selenium 0.45 ppm + zinc 60 ppm) is recommended, as it had similar effects to higher doses.

## List of abbreviations

ACE, abundance-based coverage estimator; Chao1, abundance-based estimator of species richness; CON, basal diet only; DNA, deoxyribonucleic acid; ICP-MS, inductively coupled plasma mass spectrometry; ppm, parts per million; rRNA, ribosome-ribonucleic acid; SEM, standard error of the mean value; TM, trace minerals; h, hour; min, minutes; mg, milligram.
